# Case Report: Novel likely pathogenic *MEN1* mosaic mutation in the family with MEN-1 syndrome

**DOI:** 10.3389/fendo.2025.1662871

**Published:** 2025-11-14

**Authors:** Rustam Salimkhanov, Marina Utkina, Hanum Bagirova, Anna Eremkina, Ekaterina Prosandeeva, Sergey Popov, Victoria Zakharova, Vasiliy Petrov, Alexey Novoselov, Ekaterina Bondarenko, Daria Pastukhova, Lyudmila Rozhinskaya, Natalia Mokrysheva

**Affiliations:** 1 Department of Parathyroid Pathology and Mineral Disorders, Endocrinology Research Centre, Moscow, Russia; 2 Laboratory of General, Molecular and Population Genetics, Endocrinology Research Centre, Moscow, Russia; 3 Faculty of Medicine, Lomonosov Moscow State University, Moscow, Russia; 4 Laboratory of Pathomorphology, Endocrinology Research Centre, Moscow, Russia; 5 Department of Osteoporosis and Osteopathies, Endocrinology Research Centre, Moscow, Russia; 6 Directorate, Endocrinology Research Centre, Moscow, Russia

**Keywords:** multiple endocrine neoplasia type 1, mosaic mutation, phenocopy, high-coverage NGS, genetic testing

## Abstract

Multiple endocrine neoplasia type 1 (MEN-1; OMIM 131100) is a rare, autosomal dominant syndrome caused by heterozygous inactivating mutations in the *MEN1* tumor suppressor gene (11q13; OMIM 613733). MEN-1 is characterized by polyglandular pathology, which typically involves the parathyroid glands (90%), pancreas (30-80%) and anterior pituitary (15-50%). To date, over 1,600 pathogenic *MEN1* variants have been documented, including nonsense, frameshift, and splice-site mutations, as well as rare large deletions. While germline mutation detection rates reach 70-90% in clinically diagnosed probands, approximately 10-30% of phenotypically confirmed MEN-1 families test negative by conventional sequencing, suggesting possible regulatory region defects, deep intronic mutations, or mosaic variants. In cases where *MEN1* germline testing is negative despite a clinical MEN-1 phenotype, somatic mosaicism should be considered. We investigated a familial cohort presenting with primary hyperparathyroidism, multifocal pancreatic and pituitary neuroendocrine neoplasms – a triad strongly suggestive of MEN-1. Using a multi-tissue sequencing approach, we analyzed DNA extracted from peripheral blood leukocytes and parathyroid adenomas tissue via both Sanger sequencing and next-generation sequencing (NGS) with high coverage. While conventional Sanger analysis failed to detect a mutation, targeted NGS revealed a novel, likely pathogenic *MEN1* variant present at low allele frequency (5-15%), consistent with postzygotic mosaicism. The variant was classified as pathogenic per ACMG/AMP guidelines and correlated with disease manifestations in affected tissues. These findings demonstrate that high-coverage NGS of multiple tissues is critical for identifying low-level mosaic *MEN1* mutations missed by standard testing. Alternative screening methods are required for patients with strong clinical indications of MEN-1 and/or a family history, but negative germline test results, one such method is NGS with high coverage.

## Introduction

Multiple endocrine neoplasia type 1 (MEN-1, OMIM 131100), first described by Wermer in 1954, is a rare autosomal dominant syndrome characterized by the classic triad of primary hyperparathyroidism (PHPT), pancreatic and pituitary neuroendocrine neoplasms (NENs) (1). PHPT represents the most prevalent and typically earliest manifestation, occurring in approximately 90% of MEN-1 patients by age 50 years, compared to 60% for pancreatic and 40% for pituitary NENs (2). Some patients may also develop less common or «non-classical» components of MEN-1, such as adrenocortical, carcinoid, thyroid and skin tumors (lipomas, angiofibromas, collagenomas), and others (2–4).

MEN-1 is caused by inactivating germline mutations in the *MEN1* tumor suppressor gene located at chromosome 11q13 (5). The gene comprises 10 exons encoding the 610-amino acid menin protein, which plays crucial roles in transcriptional regulation, genome stability, and cell proliferation through interactions with chromatin-modifying complexes and transcription factors (6). Identification of a pathogenic *MEN1* variant carries significant clinical implications due to the 50% inheritance risk for first-degree relatives and near-complete penetrance by age 50 years (7).

According to current international consensus guidelines (8), the diagnosis of MEN-1 can be made if at least one of the following criteria is met:

- Development of two or more «classic» MEN-1-associated endocrine tumors (parathyroid adenomas (PAs), pancreatic or pituitary NENs) (clinical criteria);
- Occurrence of one characteristic MEN-1-related tumor in a first-degree relative of a genetically confirmed MEN-1 case (familial criteria);
- Identification of a pathogenic germline variant in the *MEN1* gene (11q13), irrespective of clinical manifestations (genetic criteria).

The tumorigenesis in MEN-1 follows Knudson’s two-hit hypothesis, requiring both a germline heterozygous inactivating mutation in the *MEN1* tumor suppressor gene and subsequent somatic loss of the remaining wild-type allele through loss of heterozygosity (LOH) in target tissues (9, 10). Approximately 75% of germline *MEN1* mutations are truncating variants (nonsense, frameshift, or splice-site mutations), consistent with its tumor suppressor function (9). These mutations disrupt menin’s crucial interactions with binding partners including histone-modifying complexes, transcription factors, and DNA damage response proteins. The resultant dysregulation creates a permissive environment for clonal expansion in endocrine tissues, explaining the characteristic polyglandular involvement and tumor predisposition.

Despite meeting clinical diagnostic criteria, approximately 10-30% of familial MEN-1 cases lack detectable *MEN1* mutations by Sanger sequencing (11, 12). We present a case report of familial MEN-1 syndrome in which genetic testing revealed a mutation in the *MEN1* gene in the son but not in his father, despite the typical clinical manifestations of the disease. Further investigation revealed a novel likely pathogenic low-frequency mosaic variant of *MEN1* gene.

## Son’s clinical case description

Patient D., a 30-year-old male, was admitted to the Endocrinology Research Centre (Moscow, Russian Federation) with generalized weakness, tingling in the fingers and toes.

At the age of 8 years, D. was diagnosed with pituitary prolactinoma (hyperprolactinemia over 5000 mMU/l). A contrast-enhanced magnetic resonance imaging (MRI) of the brain revealed an endosellar pituitary microadenoma 0.4×0.35 cm. The patient was prescribed the dopamine receptor agonist cabergoline 0.5 mg weekly with positive dynamics, but without achieving remission – hyperprolactinemia resumed after discontinuation of the drug. Laboratory tests showed no abnormalities of the other hormones (thyroid-stimulating hormone, free T_4_, insulin-like growth factor 1 (IGF-1), luteinizing hormone (LH), and follicle-stimulating hormone (FSH)). The patient continues cabergoline therapy with regular blood prolactin and MRI monitoring. In 2021 (at the age of 29), normogonadotropic hypogonadism was detected (total testosterone blood level – 4.9 nmol/L, LH – 8.2 U/L, FSH – 10.1 U/L). Therapy with clomiphene 12.5 mg/day resulted in normalization of total testosterone level – 24.6 nmol/L.

Elevated PTH and blood calcium levels were first identified in May 2021 at the Endocrinology Research Centre: PTH level – 21.5 pmol/L (up to 6.9 pmol/L), hypercalcemia – 3.2 mmol/L (2.1-2.55 mmol/L), and normophosphatemia – 0.8 mmol/L (0.74-1.52 mmol/L). Daily calciuria was not evaluated. Neck ultrasound (US) showed a lesion of the left inferior parathyroid gland (PG) (3.6×1.1×1.6 cm) and a hypoechogenic nodule in the left thyroid lobe (1.2×0.9×0.7 cm, EU-TIRADS 4), consistent with the findings on contrast-enhanced multispiral computed tomography (MSCT). The blood calcitonin level was 1.0 pg/mL (up to 11.8 pg/mL). Cytology of the left thyroid nodule corresponding to a follicular neoplasm (Bethesda IV).

In August 2021, Sanger sequencing revealed a germline heterozygous *MEN1* variant c.1673_1675del (chr11:64804494_64804496del).

**Figure 1:**
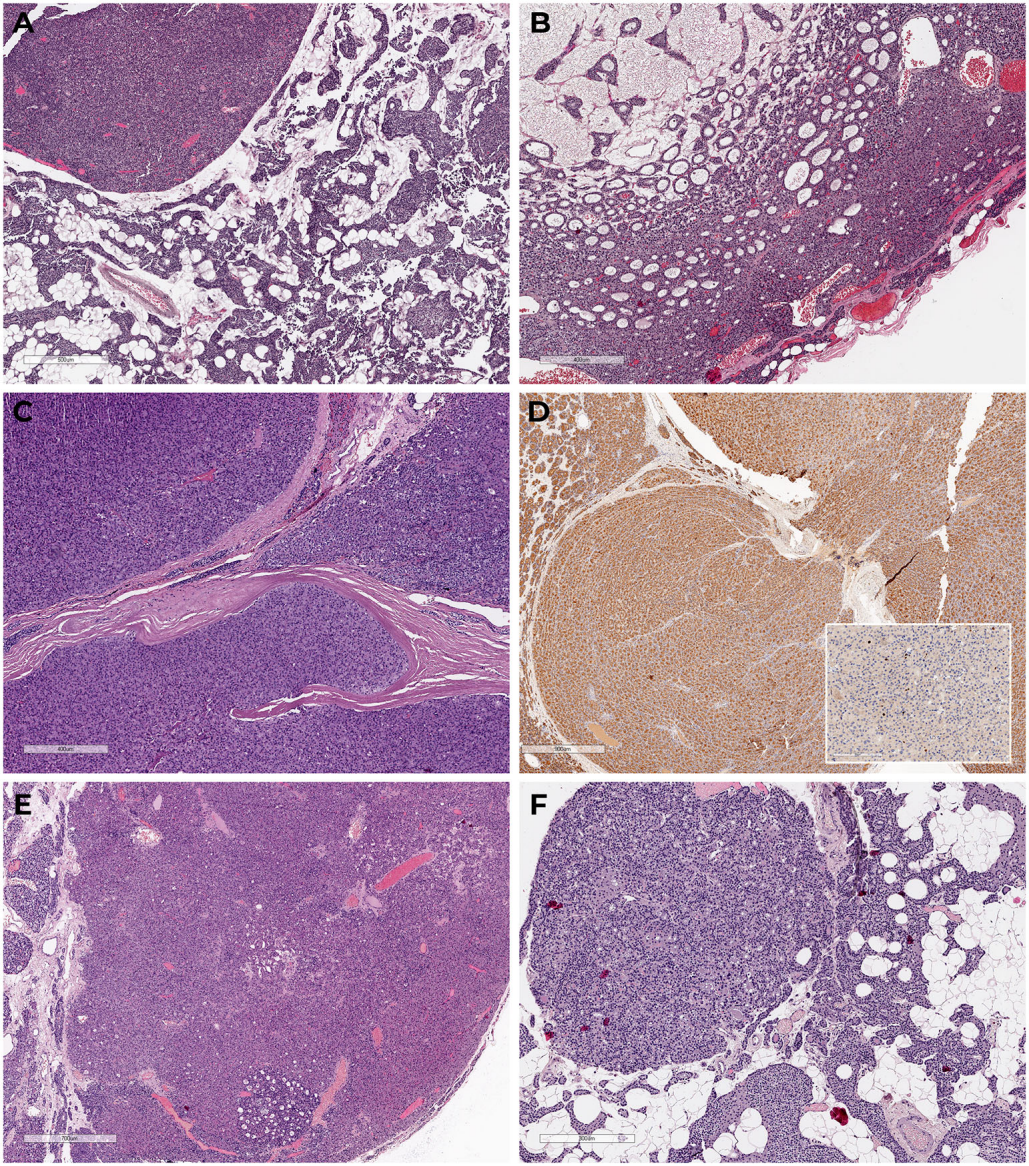
Microscopic images of parathyroid tumor’s samples **(A, B)** – PAs of left lower and upper glands from patient D, tissue was extracted after surgery in 2021 (H&E; scale bars: **(A)** – 200 μm, **(B)** – 400 μm); **(C)** – Atypical tumor of the left upper PG from patient R., tissue was extracted after surgery in 2022 (H&E; scale bar: 400 μm); **(D)** – IHC staining of the atypical adenoma of left upper PG from patient R., tissue was extracted after surgery in 2022. Diffuse PTH expression was detected in cytoplasm of tumor cells. Expression Ki-67 is shown in the right corner – 3% (scale bar: 900 μm); **(E, F)** Adenomas of the right lower and upper PG from patient R, tissue was extracted after surgery in 2022 (H&E; scale bars: **(E)** – 700 μm, **(F)** – 300 μm).

Three months later, the patient underwent parathyroidectomy with removal of two PGs (left lower and upper) with left-sided extrafascial hemithyroidectomy. Histological examination confirmed two PAs and a follicular adenoma of the left thyroid lobe (**Figures 1A, B**). Immunohistochemistry (IHC) was not performed.

Immediately after surgery, D.’s PTH blood level reduced to 25 pg/mL (15–65 pg/mL). Combined treatment with active vitamin D analogue (alfacalcidol), vitamin D (cholecalciferol) and calcium carbonate cured postoperative hypocalcemia. Dynamic laboratory tests in 2023 (at the age of 31) on calcium carbonate 500 mg/day therapy confirmed remission of PHPT: PTH level – 64 pg/mL (15–65 pg/mL) with an albumin-adjusted calcium level – 2.34 mmol/L (2.15-2.55 mmol/L).

Our patient was screened for PHPT-associated complications. Renal filtration was not impaired: eGFR (CKD-EPI) – 116 mL/min/1.73 m^2^. There was no evidence of nephrocalcinosis and nephrolithiasis on US. Dual-energy X-ray absorptiometry (DXA) scans (Z-scores) showed reduction in bone mineral density (BMD) in the femoral neck and lumbar spine (-3.0 SD and -2.6 SD, respectively). Considering the absence of low-energy fractures, no negative dynamics in BMD, and the young age of the patient, antiresorptive therapy was not prescribed. The patient continued to follow a calcium-rich diet and take calcium carbonate 500 mg daily.

In 2021, contrast-enhanced abdominal and retroperitoneal MSCT visualized a hyperintense mass (1.1x1.0 cm) of the pancreatic tail identified as NEN, which was consistent with scintigraphy (^99m^Tc-Tektrotyd) with single-photon emission computed tomography combined with X-ray computed tomography (SPECT-CT). Laboratory blood tests showed: chromogranin A – 0.8 nmol/L (up to 2.0 nmol/L), serotonin – 189.0 ng/mL (50–220 ng/mL). Laparoscopic distal hemipancreatectomy was performed in November 2021. Histology and IHC confirmed a NEN (Grade 2) with Ki-67 of 4% and positive SSTR2 and SSTR5 expression (surgery was performed at another center, histology and IHC are not available). The patient was recommended regular follow-up with imaging.

## Father’s clinical case description

Patient R., a 56-year-old male, was admitted to the Endocrinology Research Centre with severe bone pain, obesity (BMI – 30.5 kg/m^2^), and elevated blood pressure (up to 180/90 mm Hg).

R. was diagnosed with PHPT in 2021 at the age of 55. Laboratory blood tests revealed an elevated PTH level – 216 pg/mL (15–65 pg/mL), hypercalcemia – 2.7 mmol/L (2.15-2.55 mmol/L), and 25(OH)vitamin D deficiency. In December 2021, the patient underwent a right-sided nephrectomy with retroperitoneal lymphadenectomy for clear cell renal cell carcinoma (histologically confirmed, pT1aN1M0, III stage) and a staghorn stone. Neck US and dual-phase ^99m^Tc-Sestamibi scintigraphy with SPECT-CT revealed the hypoechoic mass in the left upper pole of the thyroid gland (measuring 1.2x0.6x2.0 cm). There were no signs of hyperfixation in other regions of the neck and upper mediastinum.

Sanger sequencing of the *MEN1* gene in 2022 (at the age of 56) did not detect any variants in exon 10.

In April 2022, R. underwent left upper parathyroidectomy without remission of the PHPT. Histology and IHC confirmed an atypical parathyroid tumor (**Figures 1C, D**), characterized by trabecular growth pattern, band-like fibrosis, and mitotic figures.

The further laboratory blood tests revealed increased PTH level – 141.4 pg/mL (15–65 pg/mL), hypercalcemia with a calcium adjusted for albumin – 2.6 mmol/L (2.15-2.55 mmol/L), normophosphatemia – 0.85 mmol/L (0.74-1.52 mmol/L) daily normocalciuria – 2.9 mmol/day. R. was evaluated for PHPT complications. MSCT visualized nephrolithiasis up to 3 mm in the left kidney accompanied with normal renal function (eGFR (CKD-EPI) – 87 mL/min/1.73 m^2^). DXA scans (T-scores) showed decreased BMD in the radius (-2.9 SD), lumbar spine and femoral neck (-1.9 SD and -2.4 SD, respectively). Neck US and dual-phase ^99m^Tc-Sestamibi scintigraphy with SPECT-CT visualized a tumor of the right upper PG measuring 1.7x1.0x0.6 cm.

NGS analysis of blood DNA using a custom gene panel (mean coverage: 100x) did not initially flag any pathogenic or likely pathogenic variants. However, manual inspection of the data revealed the *MEN1* variant c.1673_1675del, which was present at a low allele frequency of ~5% (5 alternate reads out of 100 total reads).

Right lower and upper parathyroidectomy was carried out in August 2022 with positive effect. Histology identified two PAs (**Figures 1E, F**). IHC was not performed. Postoperatively, the patient was prescribed alfacalcidol 1 mcg/day, calcium carbonate 1500 mg/day, and cholecalciferol 40.000 IU/week. Further follow-up confirmed the remission of the disease by 2023, therapy was gradually changed to calcium carbonate 500 mg/day with a maintenance dose of cholecalciferol. Laboratory blood tests showed normal PTH level – 45.2 pg/mL (15–65 pg/mL) and normocalcemia with calcium adjusted for albumin level – 2.2 mmol/L (2.15-2.55 mmol/L).

Contrast-enhanced brain MRI in July 2022 showed a pituitary microadenoma (0.67×0.57×0.6 cm). According to laboratory data: bioactive hyperprolactinemia – 2090 mU/L without co-secretion of IGF-1 – 213.9 ng/mL (16–245 ng/mL), endogenous hypercortisolism was excluded (daily cortisoluria – 85 nmol/day, morning blood cortisol level less than 50 nmol/L after dexamethasone 1 mg suppression test). Cabergoline 0.5 mg/week normalized a patient’s blood prolactin level. Dynamic contrast-enhanced brain MRI in July 2023 demonstrated reduced pituitary microadenoma size (5.5x4.5 mm). In July 2023, we performed a contrast-enhanced MSCT of the abdomen and retroperitoneal organs revealing adrenal nodular hyperplasia up to 8 mm without gastropancreatic NENs. The increased serum gastrin level – 881.0 pg/mL (13–115 pg/mL) was regarded as secondary to proton pump inhibitors, chromogranin A was also slightly elevated – 6.3 nmol/L (up to 2.0 nmol/L). MSCT also revealed a 59x40 mm lipoma of the right gluteus maximus muscle which was classified as a «non-classical» component of MEN-1.

## Materials and methods

Clinical data were collected retrospectively from medical records. Tumor tissue samples were collected and processed for routine histology and IHC (**Figure 1**). For IHC, 3-3.5 μm thick formalin-fixed paraffin-embedded (FFPE) sections of tumor tissue samples were prepared on adhesive slides (Menzel GmbH & Co KG, Bielefeld, Germany). Dewaxing and antigen retrieval were performed using high and low pH buffers (Leica, Wetzlar, Germany). Automated IHC staining was conducted on the BOND-III system (Leica Biosystems) using the following primary antibodies: Anti-PTH (Cell Marque MRQ-31, 1:100 dilution); Ki-67 (DAKO MIB-1, 1:100 dilution); CD34 (Leica Biosystems, QBEnd/10, ready-to-use).

All genetic testing was performed after obtaining written informed consent from the patients.

### DNA extraction and quality assessment from blood cells

Genomic DNA was isolated from peripheral blood leukocytes using the MagPure Universal DNA Kit (Magen, China) according to the manufacturer’s protocol. DNA concentration was measured using the Qubit dsDNA HS Assay Kit (Thermo Fisher Scientific, Q32851) with quantification standards ranging from 0.2 to 100 ng/μL. DNA integrity was evaluated using the Agilent 5200 Fragment Analyzer System (Agilent Technologies) with the DNA HS Kit (DNF-486, size range 1–6000 nt).

### DNA extraction and quality assessment from FFPE parathyroid tissue

Genomic DNA was extracted from FFPE tumor specimens using the QIAamp DNA FFPE Tissue Kit (Qiagen, 56404) following the manufacturer’s instructions. DNA quantification was performed using the Qubit dsDNA HS Assay Kit (Thermo Fisher Scientific). DNA quality assessment was conducted on the Agilent 5200 Fragment Analyzer using the DNA HS Kit (DNF-486), with particular attention to the degree of DNA fragmentation typical of FFPE-derived material.

### Polymerase chain reaction

Fragments of genomic DNA isolated from peripheral blood and FFPE parathyroid tissue were amplified by PCR using the following primer pairs spanning exon 10 of the *MEN1* gene: for the 731-bp fragment from blood DNA – forward 5’-ATGGCCAGAGCAGGGTC-3’ and reverse 5’-TGAGCTGGAGAAAATCGTGG-3’, and for the 292-bp fragment from FFPE DNA – forward 5’-GGACTGTCGCTGGCACCA-3’ and reverse 5’-GGTCCGAAGTCCCAGTAGTT-3’. The reaction mixture contained 1× PCR mix (Eurogen, Russia), 5 μM each of forward and reverse primers, and 1–20 ng of genomic DNA in a final volume of 20 μL. Amplification was performed in a VeritiPro Thermal Cycler (Applied Biosystems) under the following conditions: initial denaturation at 95°C for 5 min; 10 cycles of 95°C for 15 sec, 65°C for 20 sec, and 72°C for 40 sec; followed by 25 cycles of 95°C for 15 sec, 60°C for 20 sec, and 72 °C for 40 sec; with a final extension at 72°C for 3 min.

### Sanger sequencing

Using PCR, fragments of genomic DNA isolated from blood and FFPE tissue were amplified, targeting the 10th exon of the *MEN1* gene. PCR products were purified using ExoI (1.7 U) and FastAP (0.1 U) enzymes. Cycle sequencing was performed using the BigDye Terminator v3.1 Cycle Sequencing Kit (Applied Biosystems) according to the manufacturer’s protocol. Sequencing products were analyzed by capillary electrophoresis on the Genetic Analyzer 3500 (Applied Biosystems). Sequence alignment and analysis were performed using two independent methods: electronic analysis with a 10% detection threshold and manual visual inspection of electropherograms using Sequencher software (Gene Codes Corp., Inc.).

### DNA sequencing and analysis

We employed a custom-designed NGS enrichment panel (Endome V2) covering the coding regions of 377 genes. DNA libraries were prepared using the KAPA HyperPlus Kit (Roche) following the manufacturer’s protocol, with subsequent sequencing performed on an Illumina NextSeq 550 platform using the NextSeq 500/550 Mid-Output Kit v2.5 (300 cycles) to generate 150 bp paired-end reads. Initial quality control was conducted using fastp (v0.23.2), followed by alignment of processed fastq files to the GRCh38 human reference genome (GATK best practices) with BWA-mem (v2.2.1). Post-alignment processing included duplicate removal and sorting using samtools (v1.9). Comprehensive analysis of sorted BAM files involved: (1) coverage assessment via mosdepth (v0.3.3) at X1/X10/X20/X50/X100 thresholds using panel-specific BED coordinates; (2) variant detection with deepvariant (v1.4.0); and (3) variant quality filtration employing BCFtools (v1.18) according to established parameters.

### Genetic studies results

**Figure 2:**
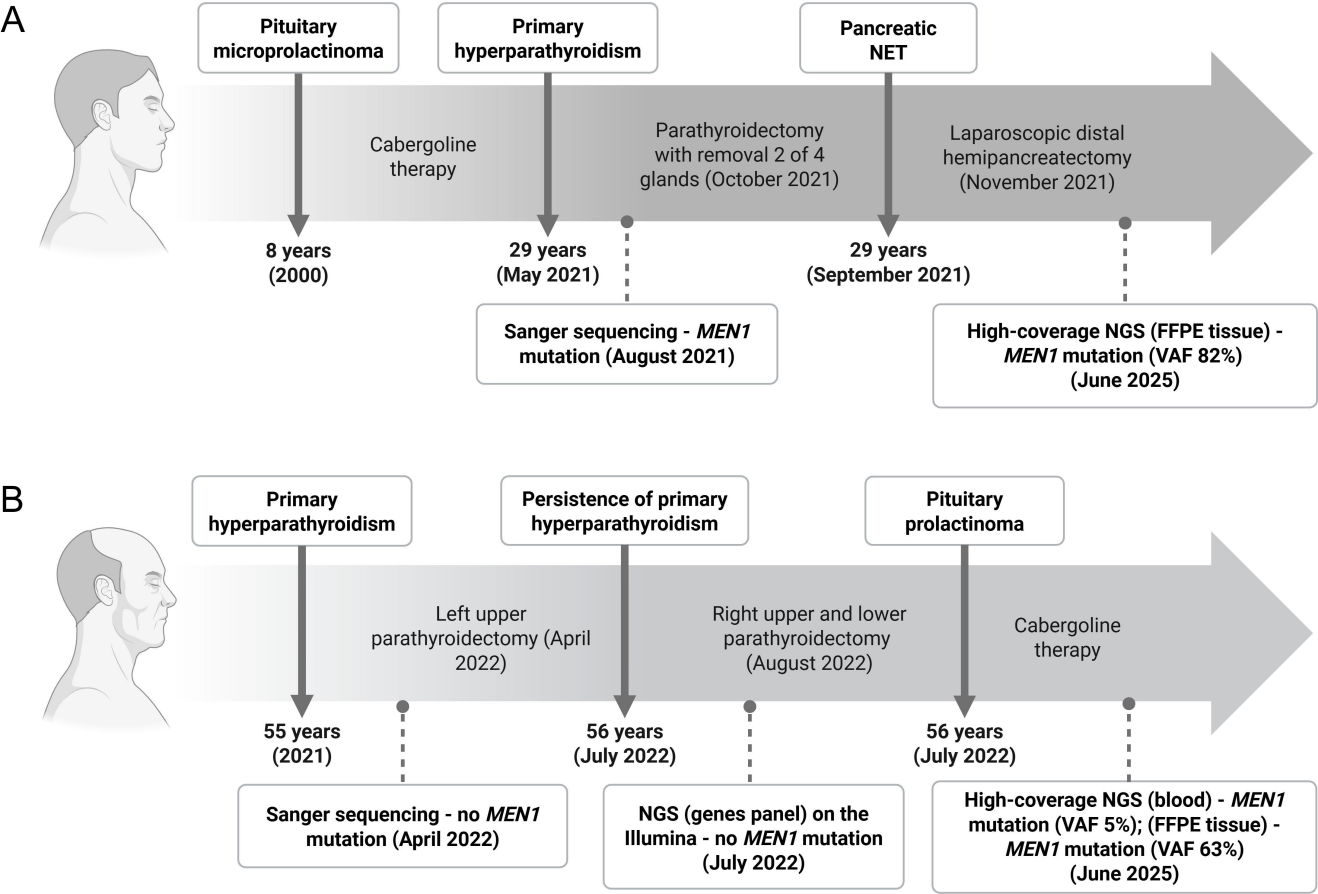
Timeline of main MEN-1 clinical events in son **(A)** and father **(B)** with genetic testing steps, including Sanger sequencing, NGS, and high-coverage NGS (created with BioRender).

**Figure 3:**
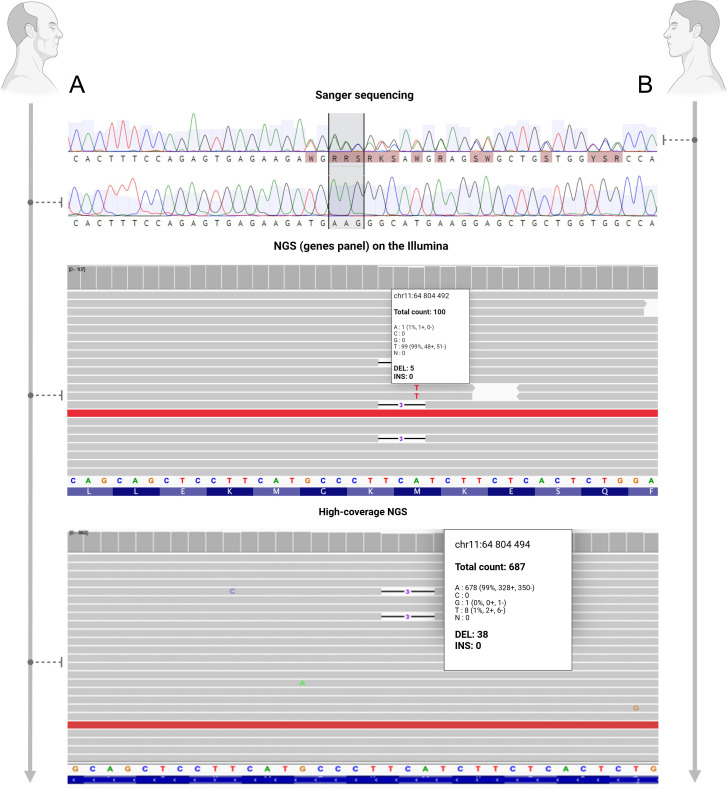
Analysis of the exon 10 sequence of the *MEN1* gene (NM_001370259.2) in the blood cells of patients **(A)** and **(B)** Sanger sequencing and high-coverage NGS revealed a heterozygous mutation c.1673_1675del (chr11:64804494_64804496del) in patient **(A)** The deletion causes a frameshift, visible on the Sanger chromatogram as overlapping peaks starting at the mutation site (indicated by the gray area). The number of reads supporting the deletion is shown using the first nucleotide as an example. The figure was created with BioRender.

Sanger sequencing of leukocyte DNA from patient D. (son) identified a heterozygous likely pathogenic *MEN1* variant (NM_001370259.2:c.1673_1675del, p.Met558del) in exon 10 (**Figures 2D**, **3D**), while analysis of patient R. (father) failed to detect this variant despite his son’s positive result (**Figures 2R**, **3R**). Subsequent NGS using the Endome-V2 panel (covering 27 endocrine tumor-associated genes including *MEN1*, *CASR*, *CDC73*, *AIP*, *GNAS*, *PRKAR1A*, and *SDHx* family) achieved 103× average coverage with 99% of targets >10× using 2×150 bp paired-end sequencing on Illumina NextSeq (**Figures 2R**, **3R**).

**Figure 4:**
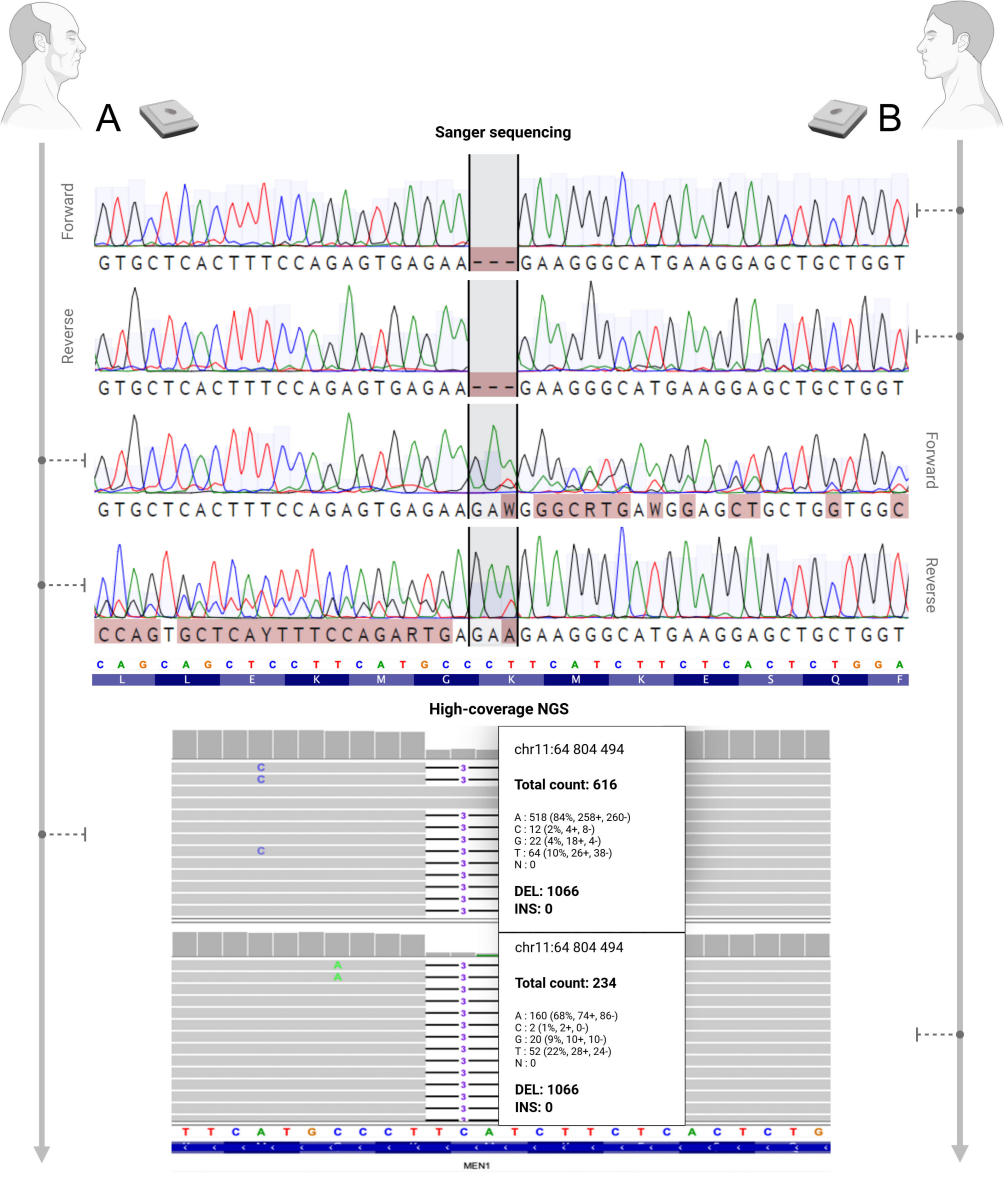
Analysis of exon 10 of the *MEN1* gene (NM_001370259.2) in FFPE samples from the PGs of patients **(A)** and **(B)**. In patient **(B)**, Sanger sequencing and high-coverage NGS revealed the c.1673_1675del (chr11:64804494_64804496del) mutation in a hemizygous state, consistent with somatic LOH of the wild-type allele in the tumor tissue. The Sanger chromatogram shows a clean, shifted sequence without overlapping peaks. In patient **(A)**, a heterozygous c.1673_1675del mutation was confirmed. The Sanger chromatogram displays overlapping peaks starting at the mutation site, indicating the presence of both wild-type and mutant alleles. Chromatograms for forward and reverse strands are shown for each patient. The number of NGS reads supporting the deletion is shown using the first nucleotide as an example. The figure was created with BioRender.

The clinical presentation of MEN-1 in patient R. (father) likely corresponds to his son’s confirmed *MEN1* mutation, prompting high-coverage NGS analysis of exon 10 (731 bp amplicon) in both individuals using MiSeq sequencing with GRCh38 alignment. Patient D. (son) exhibited 82% variant allele frequency (VAF) (1066/1300 reads) for the c.1673_1675del (p.Met558del) mutation in FFPE tissue, confirming heterozygosity (**Figures 2D**, **3D**, **4D**). Patient R.’s (father) blood-derived DNA showed low-level mosaicism (5% VAF, 38/725 reads), while FFPE tissue analysis revealed predominant mutation presence (63% VAF, 1066/1682 reads) (**Figures 2R**, **3R**, **4R**), demonstrating tissue-specific mutational burden consistent with postzygotic mosaicism.

This analysis identified a likely pathogenic mosaic variant c.1673_1675del, p.Met558del in exon 10 of *MEN1* in R. For clarity, we refer to this variation as «*MEN1* mosaic».

## Discussion

Genetic testing is currently the preferred method for confirmation of MEN-1. Searching for the *MEN1* gene mutation in relatives allows timely diagnosis regardless of clinical presentation, especially since no definitive genotype-phenotype correlation has been observed for this syndrome (13). This approach can be used to expand testing of laboratory and instrumental MEN-1 components and develop effective follow-up plans (14).

More than 30% of patients with classic clinical manifestations of MEN-1 do not have an identified *MEN1* gene mutation in the coding region and splice sites, in these cases, further testing such as partial or complete deletion testing, *MEN1* locus haplotype analysis, or additional gene analysis should be considered (7, 14). *MEN1* gene mutations are spread throughout the coding region without specific «hot spots». To date, more than 1600 germline *MEN1* mutations have been identified (9, 15). Moreover, *de novo* mutations of the *MEN1* gene account for over 10% and can be inherited (Marini et al., 2006).

Patients with MEN-1 phenotype, but without *MEN1* mutation considered as a phenocopies (16). Mutations in the *CDKN1B* gene may be associated with MEN-1-like syndrome – MEN-4. Mutations in other genes, including *CaSR*, *AIP*, and *CDC73*, can also cause phenotypic manifestations similar to MEN-1. Phenocopies could potentially explain the simultaneous occurrence of two endocrine disorders in a single patient, each with a different etiology (16).

Mosaicism, a phenomenon in which genetic variations are acquired spontaneously during cell division in postzygotic embryonic development, may be associated with inherited syndromes (17–19). Mosaicism may be one of the main causes of unsolved cases in patients with the classic clinical features of MEN-1, when traditional genetic sequencing techniques fail to detect mutations. To date, only a few cases of *MEN1* mosaicism have been described in the literature (20–26). Clinical manifestations in mosaic patients may differ depending on embryonic development and the types of mutated cells (27). Patients with mosaicism can pass the mutation to their offspring depending on the proportion of mutated germ cells. Some studies have suggested a milder phenotype of MEN-1 in mosaic patients, but this view has not been confirmed. As in our case, mosaic MEN-1 patients can develop the classic triad of components, characterized by a clinically aggressive course and early manifestation of the syndrome (24, 26). Patient D. (son) developed all three classic components of the syndrome (early onset of PHPT, pituitary and pancreatic NENs), corresponding to a higher VAF. Patient R. (father) had a milder phenotype of MEN-1 – only 2 major components of MEN-1 (PHPT with multiglandular PG’s involvement and pituitary microadenoma were diagnosed by age 56).

The most commonly used methods for detecting mutations in the *MEN1* gene are Sanger sequencing and NGS. Both approaches are valuable, but they vary in technique and data processing. While Sanger sequencing remains the «gold standard» for accuracy, its utility is constrained by low throughput, high costs for parallel multi-gene analysis or large sample sets, and limited sensitivity in detecting mosaic variants (28). This method is suitable for studying a known mutation or searching for mutations in a known region of the genome. Unlike Sanger sequencing, NGS is a more advanced technique that allows millions of DNA fragments to be sequenced in parallel, enabling much faster and more effective genome sequencing. In addition, NGS is able to detect structural differences and single nucleotide polymorphisms within the genome. It is used in genomics, mutation research, and the diagnosis of genetic diseases, such as MEN-1 (29).

While the mutation in patient D. (son) was detected in the germline DNA in a heterozygous form, the tumor tissue exhibited a hemizygous state for this variant (**Figure 4D**). This could be due to allelic loss, where somatic inactivation of the wild-type copy of the *MEN1* gene occurs. In 1993, Knudson suggested that MEN1-related tumors (as well as some other tumor types associated with tumor suppressor genes) require inheritance of a germline mutation along with a somatic mutation in the tumor DNA, leading to LOH (9, 30). Various cases have been identified in benign nodules and carcinomas of the thyroid and PG’s, where heterozygous *MEN1* patients exhibited allelic loss of the wild-type allele at the 11q13 region (31–33). In addition, many sporadic cases of parathyroid and thyroid gland tumors in which 11q13 LOH was observed have been reported (34–36).

In our study, none of the previously mentioned methods were able to detect the mosaic mutation. As a result, we decided to use NGS with high coverage to identify the unknown mosaic mutation. We consider that physicians and geneticists should keep in mind that mosaicism is likely underestimated in unresolved cases of MEN-1 patients.

## Conclusion

These cases illustrate the potential for germline mutations to be missed by routine screening and highlight the importance of considering mosaicism in cases where mutations are not detected. It is therefore important to discuss the benefits of performing additional genetic analyses, such as searching for mosaicism, copy number variation, deep intronic variants, or re-analysis using different bioinformatics software, in cases where a patient with a phenotype suggestive of MEN-1 has a negative genetic result for *MEN1*. These diagnostic difficulties should be discussed on a case-by-case basis between molecular scientists and clinicians.

## Data Availability

The original contributions presented in the study are publicly available. This data can be found here: https://www.ncbi.nlm.nih.gov/sra/PRJNA1212883.
